# Injectable Biocomposites for Bone Healing in Rabbit Femoral Condyle Defects

**DOI:** 10.1371/journal.pone.0075668

**Published:** 2013-10-16

**Authors:** Jianheng Liu, Kezheng Mao, Zhengsheng Liu, Xiumei Wang, Fuzhai Cui, Wenguang Guo, Keya Mao, Shuying Yang

**Affiliations:** 1 Department of Orthopaedics, General Hospital of People's Liberation Army, Beijing, China; 2 Department of Materials Science and Engineering, Tsinghua University, Beijing, China; 3 Beijing Olympic fine Pharmaceutical Technology Co., Ltd, Beijing, China; 4 Department of Oral Biology, University at Buffalo - SUNY, Buffalo, New York, United States of America; University of Notre Dame, United States of America

## Abstract

A novel biomimetic bone scaffold was successfully prepared in this study, which was composed of calcium sulfate hemihydrate (CSH), collagen and nano-hydroxyapatite (nHAC). CSH/nHAC was prepared and observed with scanning electron microscope and rhBMP-2 was introduced into CSH/nHAC. The released protein content from the scaffold was detected using high performance liquid chromatography at predetermined time interval. In vivo bone formation capacity was investigated by means of implanting the scaffolds with rhBMP-2 or without rhBMP-2 respectively into a critical size defect model in the femoral condyle of rabbit. The releasing character of rhBMP-2 was that an initial burst release (37.5%) was observed in the first day, followed by a sustained release and reached 100% at the end of day 20. The CSH/nHAC showed a gradual decrease in degradation with the content of nHAC increase. The results of X-rays, Micro CT and histological observation indicated that more new bone was formed in rhBMP-2 group. The results implied that this new injectable bone scaffold should be very promising for bone repair and has a great potential in bone tissue engineering.

## Introduction

Nowadays, the number of patients suffering from bone tumor resections, fracture defects, or chronic infection is increasing rapidly owing to disease and trauma, and more than 1 million bone graft operations are performed in the United States every year [1]. For the treatment of nonunion and bone defects, autograft is the gold standard for bone repair. However there are some disadvantages associated with the autografts, such as the limited abundance in supply, new nerve damage, persistent pain and new fractures. Allografts have been used successfully in the orthopedic operations owing to its excellent osteoconductivity and abundance in supply. However, allografts have the potential risk of infecting, disease transmission and immune response. On the other hand, allografts are inferior in promoting bone regeneration compared to autografts, because it required processing, sterilization steps, and preservation before it used [2]–[5]. Thus, tissue engineering might provide with a promising approach for bone regeneration [6]. An ideal bone grafting material should not only provide mechanical strength, a void space for vascularization and tissue infiltration but also be both osteoinductive and osteoconductive, and serving as a carrier for relevant therapeutical factors [7], [8]. At present, we have various biomaterials for bone regeneration, such as bone-derived collagen, decalcified bone matrix, fibrin, nano-hydroxyapatite/collagen, synthetic poly (glycolic-co-lactic acid) polymer, true bone ceramics or sintered bovine bone, and titanium [9]–[14]. Each has its own advantages, at the same time a variety of disadvantages still remain. For example, ceramic and polymer-based bone graft substitutes are mostly osteoconductive but deficiency in osteoinductivity. Other problems may include unsuitable degradation rates and inferior mechanical properties. In addition, protein- or growth factor based bone graft substitutes usually needs an addition of an osteoconductive scaffold for structural support [5], [15]. Previous studies have revealed that natural bone is a 3-demension composite which has an intricate hierarchical structure of mineralized collagen fiber by nano-hydroxyapatite crystals [16]. Inspired from this, many researchers have fabricated nano-hydroxyapatite/collagen (nHAC) by biomimetic strategy and it shows great promise in clinical applications because its compositional and structure are similar to natural bone [17], [18]. We also know that calcium sulfate hemihydrate (CSH) is a degradable and nontoxic bone repair materials and it has been widely used in the clinic as a bone regeneration scaffold due to its rapid setting and good biocompatibility properties [19]–[21]. In the operation, the surgeon has to fit the surgical site around the implant because of lacking of available implants with a desired shape. This condition can lead to increased bone loss, trauma to soft tissue, and a longer time for the operation [22]. In this study, a novel bone tissue engineering substitute composed of CSH and nHAC has been fabricated successfully, which aims to have complementary advantages for the nonloading bearing bone defect repair in orthopedic application [23]. CSH/nHAC is an injectable bone graft material and has self-setting property. Besides these, this biocomposite has good biocompatibility and strong ability to accelerate the formation of new bone [24]. Some other relevant results indicate that CSH/nHAC exhibits better bioactivity and favorable biocompatibility, and this composite can improve cell attachment and stimulates cell proliferation and differentiation [23], [24]. Since Urist used demineralized bone matrix to induce ectopic bone formation in 1965, the experimental studies on bone morphogenetic proteins has become a focus of investigation in orthopaedic surgery [25], [26]. Some experiments reveal that bone morphogenetic proteins play a critical role in cell differentiation, cell proliferation, during the process of bone and cartilage formation [27], [28]. Recombinant human BMP-2 (rhBMP-2) has already been clinically applied to induce bone regeneration in fracture healing and spinal fusion [29], [30]. Biomaterials combined with BMP-2 has shown the ability to heal some bone defect in animal model [31], [32]. The induced bone formation ability of rhBMP-2 makes it the most powerful BMP [15], [33].

In the present experiment, CSH/nHAC composite loaded with or without rhBMP-2 was prepared. In vivo bone formation capacity was investigated by means of implanting the scaffolds into a critical size defect model in the femoral condyle of rabbit. In vitro release kinetics and degradation characteristics of CSH/nHAC were also studied.

## Materials and Methods

### Ethics

The use of laboratory rabbits and the surgical procedures were according to the policies and principles established by the Animal Welfare Act and the NIH Guide for Care and Use of Laboratory Animals. The PLA General Hospital Animal Care Committee approved all the experimental procedures(#SCXK2012-0001). Throughout surgery, each rabbit was preoperatively anesthetized with an intramuscular injection of sodium pentobarbital (25 mg/kg) and xylazine(8 mg/kg) for pain management. Animals were monitored once daily immediately after surgery and then 3–4 times per week. During the study, humane endpoints were used in accordance with PLA General Hospital standard operating protocol. In case of infection at the surgical site, wound dehiscence, weight loss (>20%) or if the animal became cachectic, had difficulty eating, drinking or moving around freely, the animal was euthanized. The rabbits were euthanized by CO_2_ asphyxia under general anesthesia at the time of sacrifice. This method is consistent with AVMA (American Veterinary Medical Association) euthanasia guidelines on the use of CO_2_ as a euthanizing agent.

### Material preparation and characterization

Calcium sulfate dihydrate (CSD), medicine grade, was obtained from Merck, (Germany). rhBMP-2 was purchased from Pepro Tech Asia, Ltd. (China). nHAC was prepared by self-assembly of nano-hydroxyapatite and collagen [34]. α-calcium sulfate hemihydrate (CSH) were prepared by hydrothermal synthesis of CSD in the Department of Materials Science and Engineering of Tsinghua University [35]. Synthesis of CSH/nHAC scaffold was prepared as previously reported [23]. In the animal experiment, CSH were mixed with nHAC at the content of 10% (weight ratio). The mixed powders were moistened by deionized water. The liquid to powder (L/P) ratio was 0.8 mL/g. After final setting at 37°C for 24 h, the phase composition of prepared samples was characterized by X-ray diffraction (XRD, D/max-2500×) using monochromated Cu Kαradiation (λ = 1.5405E, 120 mA, 40 kV) in a continuous scan mode(Scanning speed, 8°/min; 2θrange,10° to 60°). Use scanning electron microscopy (SEM, JSM6460, JEOL, Japan) to detect the morphology of prepared materials.

### Preparation of Implants

For the preparation of an injectable biocomposite, 10 µg rhBMP-2 was dissolved in a total volume of 10 ml saline and CSH/nHAC(2 g) was mixed with 1 ml saline described above. The biocomposite was sterilized with ethylene oxide gas before mixed with saline. CSH/nHAC and rhBMP-2 were aseptically mixed in a dish in the operation.

### *In vitro* Release Assay

The CSH/nHAC scaffolds with rhBMP-2 (1 µg/g) were prepared as cylinder and stored at 4°C for 24 h. Scaffolds preprocessed with equal volumes of saline were used as controls. The samples were incubated in 5 ml simulated body fluid (SBF) in a shaking incubator at 37°C for 25 days(n = 6). The SBF was prepared according to the procedure as previously described [36]. The ion concentrations of SBF are similar to that in human serum. The release of rhBMP-2 was examined at 2 h, followed by 1, 2, 3, 5, 7, 10, 12, 14, 16, and 20 days. At each time interval, the supernatant was removed completely with fresh buffer added. The amounts of the rhBMP-2 in the collected supernatants were measured by high performance liquid chromatography (HPLC). Calculate the cumulative release amounts of rhBMP-2.

### *In vitro* Degradation

The degradation of CSH/nHAC and control CSH were performed in SBF. Briefly, the CSH/nHAC scaffolds with different content of nHAC, the CSH/nHAC scaffolds with or without rhBMP-2 and CSH scaffolds were placed individually into 15 ml tubes containing 10 ml SBF. The scaffold containing tubes were then placed in a 37°C shaking water bath at 100 rpm for 1, 2, 4 and 8 weeks. The SBF solution was changed every 7 days. At the predetermined time points, samples were removed from SBF and rinsed with DI water for two times. At last, the collected samples were lyophilized for a week to ensure complete removal of water. Then the lyophilized samples were weighed. The degradation ratios of the scaffold were measured. The change of scaffold mass was recorded as percentage mass remaining as calculated from the equation below:
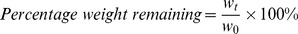
Where w_t_ represents the mass of scaffold at the predetermined time and w_0_ standard for the initial scaffold mass.

### Animals and Surgical Procedures

**Figure 1:**
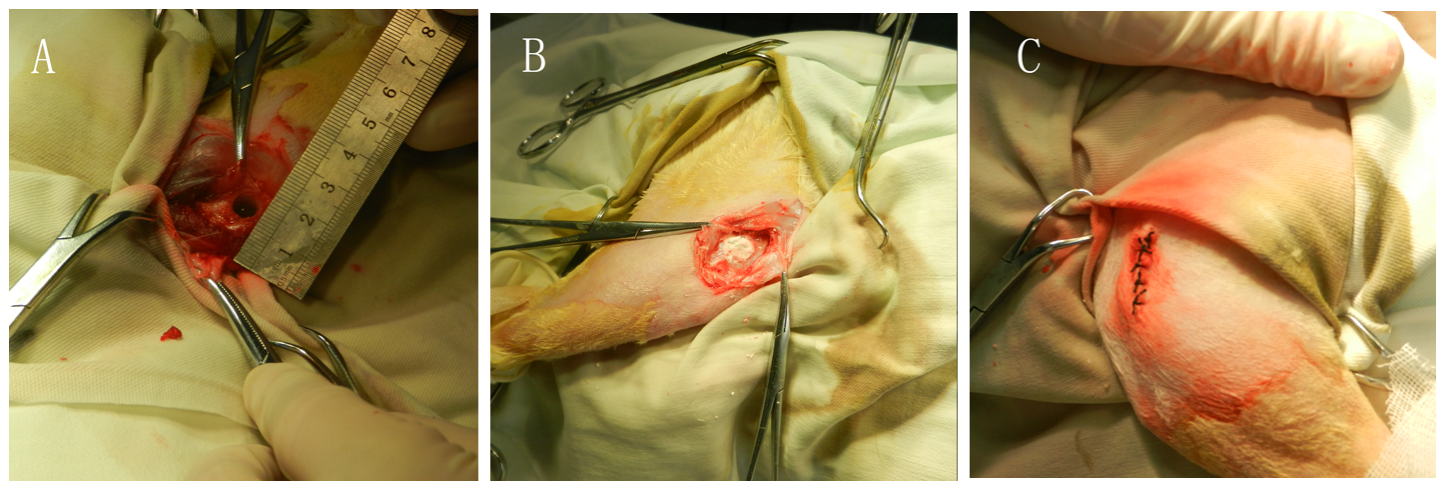
Macroscopic appearance of femoral condyle bone defect (A); Defects were implanted with bone scaffolds (B); the appearance of the surgery site after operation (C).

The use of laboratory rabbits and the surgical procedures were according to the policies and principles established by the Animal Welfare Act and the NIH Guide for Care and Use of Laboratory Animals. Throughout surgery, each rabbit was preoperatively anesthetized with an intramuscular injection of sodium pentobarbital (25 mg/kg) and xylazine(8 mg/kg) for pain management. Thirty-six male New Zealand White rabbits weighting 3.0–3.5 kg offered by the Experimental Animals Center of PLA hospital were used for this experiment. The experiment animals were divided into three groups depending on the implants placed in the bone defects: A, the control group; B, CSH/nHAC group; C, CSH/nHAC/rhBMP-2 group. Twelve randomly selected rabbits were assigned to each study group. The femurs of each rabbit were clipped and scrubbed with povidone-iodine and a 75% ethanol solution. A 5 cm longitudinal skin incision was made in the lateral femoral condyle of rabbit. Skin and musculature were then dissected. Then the femoral condyle was exposed. A critical size defect model were created using a dental burr in the middle of femoral condyle (7 mm in diameter,10 mm in depth)[Figure 1(A)]. Then the bone scaffolds were implanted into the bone defect area[Figure 1(B)]. At last, repair the muscle attachment and close the skin in layers [Figure 1(C)]. We did not use fixation device in this experiment. All the animals received an intramuscular injection of antibiotics for three days after surgery. The rabbits were kept in the cage freely and given their traditional regimen of food and water after operation.

### Radiographic Examination and Micro CT Evaluation

For radiographic evaluation and histological examination, four animals from each group were sacrificed by overdose of pentobarbital at 4, 8, 12weeks after surgery. After removing the soft tissue attached to the femur, the rabbit femurs were examined by X-rays (Siemens, Germany) and Micro CT (ZKKS-MCT-Sharp, China). The X ray images were graded and analyzed statistically. The formation of mineral in the bone defect area was evaluated with planimet. The area of the defect that was occupied by bone was quantitated from X ray images and recorded as a percentage of the total area of the defect for each sample at each time internal. Bone formation was scored on a 6-point scale as follows: 0, no formation; 1, formation of less than 25 percent; 2, formation of more than 25 percent; 3, formation of more than 50 percent; 4, formation of more than 75 percent; 5, formation of 100percent. Four obsevers independently scored the bone formation in each defect [31].

### Histology

After the radiographic examination and Micro CT evaluation, the harvested femur were washed with saline thoroughly, then fixed in 4% paraformaldehyde and subsequently decalcified for 5 weeks in 10% EDTA, PH 7.0 at 4°C. After dehydration and complete decalcification, the samples were embedded in paraffin wax. Then 5 µm vertical serial slices were prepared using a microtome, subsequently surface staining was performed with haematoxylin and eosin (H&E), Sirius red and Toluidine blue staining for microscopic observation.

### Histomorphometry

To compare the efficiency of bone regeneration in quantity, semi-quantitatively analysis on histological sections with H&E staining and Sirius red staining were performed. Images of the sections were taken with an Olympus BX51 light microscope connected with a charge coupled device (CCD) camera. For H&E staining, four pieces of histological sections were chosen in the middle of femoral condyle in the sagittal plane from three groups at different time intervals and observed under microscope at 10X magnifications. Choose 5 images randomly in the same section. Analyze the images by image software Image-Proplus (Media Cybernetics, USA). New bone formation was expressed as a percentage, and that is the new formed bone area accounts for the original bone defect area, which can be described in the equation below:
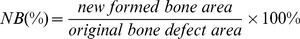
For Sirius red staining, four pieces of histological sections were chosen in the middle of femoral condyle in the sagittal plane from these three groups at different time points and observed under microscope at 10X magnifications. At least 5 images were randomly chosen in the same section and analyzed by Image-Proplus software. Calculate the type I collagen area within the original defect area.

### Statistical Methods

The data collected in the experiment were expressed as means±standard deviation(SD). Statistical analyses of the data were performed using ANOVA and the post hoc Tukey's test. The difference was regarded statistically significant when *p*<0.05.

## Results

### Characterization of the CSH/nHAC composite

**Figure 2:**
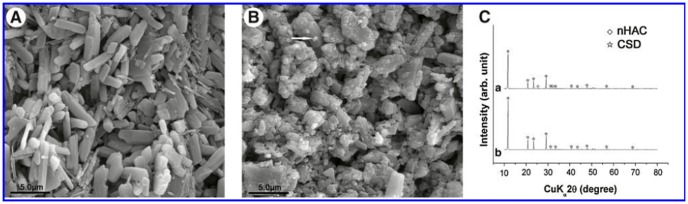
SEM micrographs of CSH crystal (A), CSH/nHAC (B), XRD patterns for composite (CSH/nHAC 10%).

XRD was used for detecting the composition of this bone scaffold. The diffraction results of the CSH/nHAC composite prepared in this experiment were same as previously described [37]. CSD appeared owing to the hydration of CSH [24]. Figure 2 shows SEM micrographs of CSH and nHAC. CSH was found to have a rod-like crystal structure and nHAC was found that it was composed of many irregular particles [Figure 2 (A, B)]. The main composition of the final setting composite is CSD and nHAC [Figure 2 (C)]

### *In vitro* rhBMP-2 Release

**Figure 3:**
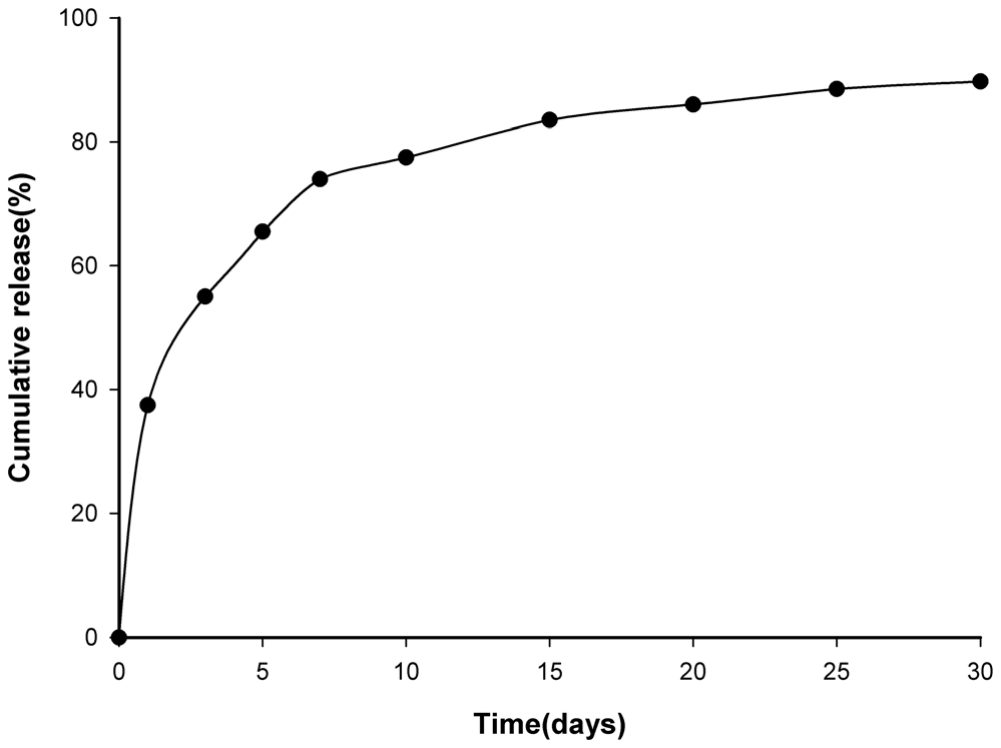
The cumulative release percentage of rhBMP-2 from the CSH/nHAC composite.

The cumulative release amounts of rhBMP-2 from the CSH/nHAC scaffold were measured and depicted in Figure 3. The release rate was very rapid on the first day, and approximately 37.5% of rhBMP-2 had been released from the CSH/nHAC. Then the rhBMP-2 was released in a sustained way, but the release rate decreased significantly with time. By day 20, approximate 100% of the total rhBMP-2 had been released from the materials.

### *In vitro* degradation of the CSH/nHAC composite

**Figure 4:**
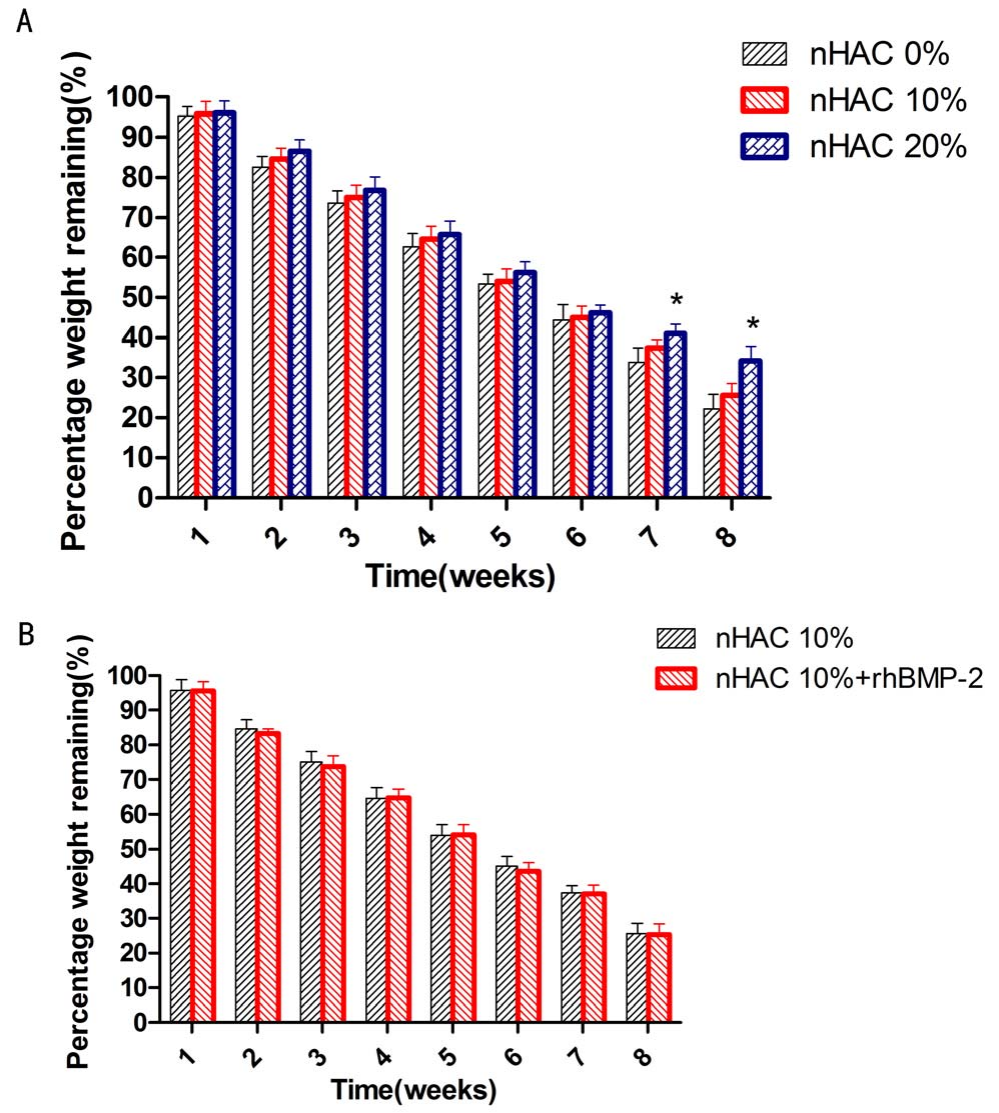
Degradation of the CSH/nHAC scaffold with different content of nHAC for various times (A); Degradation of CSH/nHAC composites (10%nHAC) loaded with or without rhBMP-2(B). *Statistically significant difference between the degradation of the composite CSH/nHAC (20% nHAC) and CSH (n = 5, *p*<0.05) (A).

Degradation rate of the CSH/nHAC scaffold with different content of nHAC was shown in Figure 4(A). It shows that there was no significant difference of the degradation rate between the CSH/nHAC scaffold and the CSH at the early 6 weeks. At the end of 7th and 8th week, CSH/nHAC with 20% nHAC degraded significantly slower than the CSH. As is reported before, the degradability of a biomaterial has a vital effect on the performance of bone regeneration. In this experiment, we also discuss the effect of adding rhBMP-2 on the degradability of CSH/nHAC. Figure 4(B) characterizes the degradability of CSH/nHAC (10%) with or without rhBMP-2 for various times. We can see that the composites degraded gradually in SBF solution, and the degradation rate had no significant difference with the addition of rhBMP-2.

### General Conditions of Animals

All animals woke up one hour after the operation, and could stand up in the first day and walk freely two days after operation. One rabbit in Group B died three weeks after surgery because of the bone fracture at the defect site. The other rabbits showed an undisturbed wound healing and did not have any clinical signs of inflammation at the surgical sites. The ROM (range of motion) of knee joints of rabbits was almost normal. The skin of the incision area showed no swelling or redness.

### Radiographic Examination

**Figure 5:**
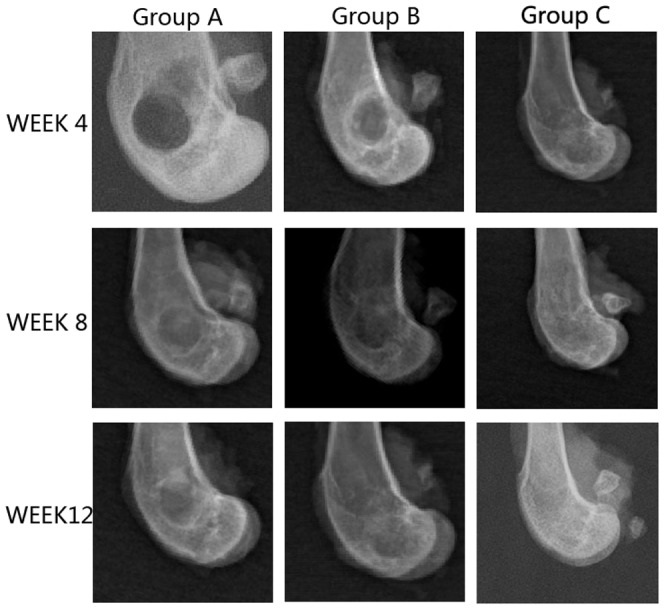
Radiographic images of defect site of three groups.

After 2 weeks of operation, new bone formation was not observed in all samples. At 4 weeks after the operation, the margins of the scaffolds were becoming unclear and some high-density signal emerged at the edge of the bone defect indicated that new bone was forming at the bone defect area in group B and C. At 8 weeks after surgery, most of the implanted materials had disappeared. Bone implanted materials have been replaced by new formed bone which filled in the bone defects partially. Most of the regions in bone defect area could not be observed in groups B and C. Some samples of group C even repaired completely. At 12 weeks, the boundary between the newly formed and host bone disappeared and complete healing of the bone defect was observed in all 12 rabbits in group C. In group B, the bone defects did not repair as better as group C. However, no bone formation was seen at up to 8 weeks after operation and only a small amount of bone formation was observed in the bone defect area at 12 weeks in group A [Figure 5]. Besides this, we also got X ray score results. After 12 weeks, there were 8 samples above 4 point in group C. For group A and group B, it was 0 and 3. Significant differences were observed when the three groups were compared in terms of bone formation. The rhBMP-2 group had significantly more bone formation than the other two groups(p<0.05).

### Micro CT Evaluation

**Figure 6:**
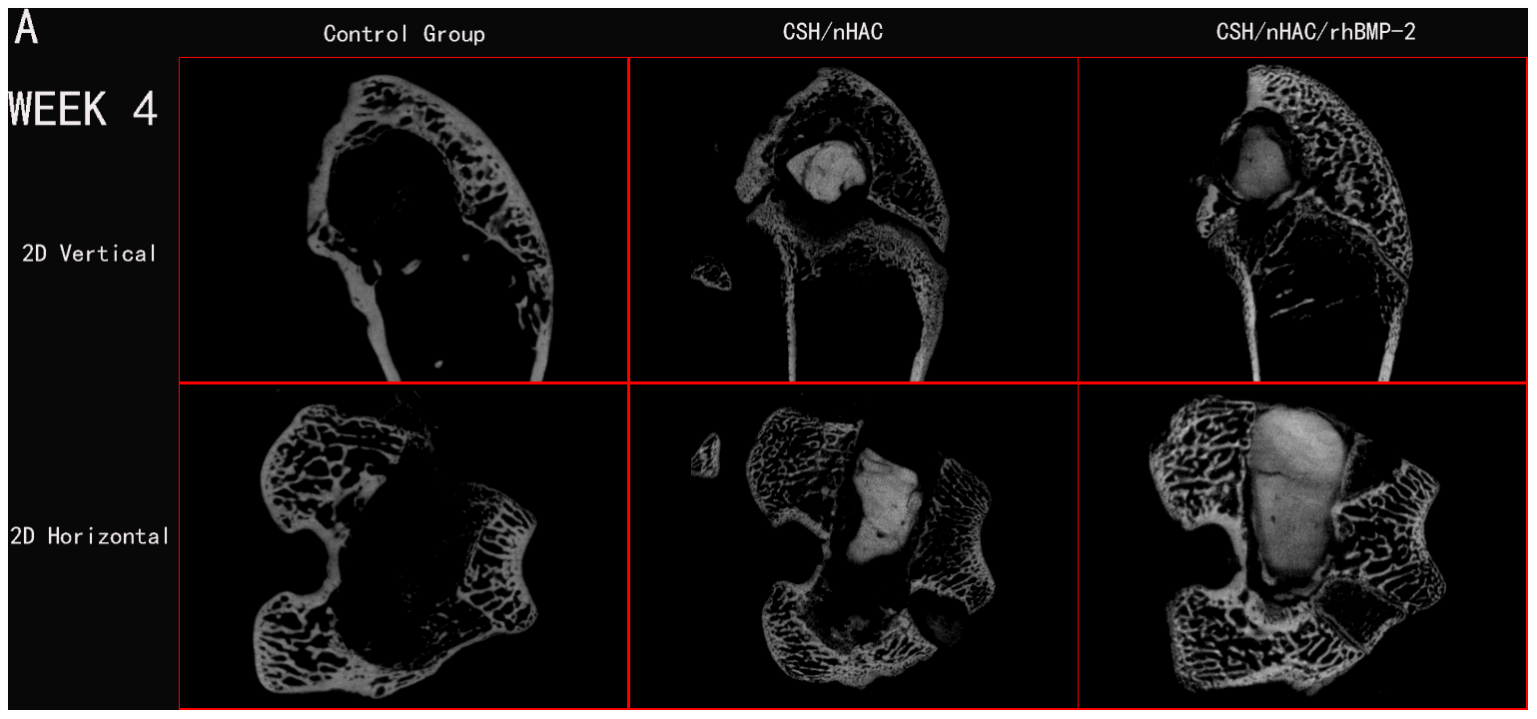
In vivo evaluation of the effects of CSH/nHAC scaffolds on new bone formation in a rabbit femoral condyle bone defect model. Treatment groups included: control group, CSH/nHAC group, CSH/nHAC/rhBMP-2 group. And the harvested Micro CT images for 4 weeks.

**Figure 7:**
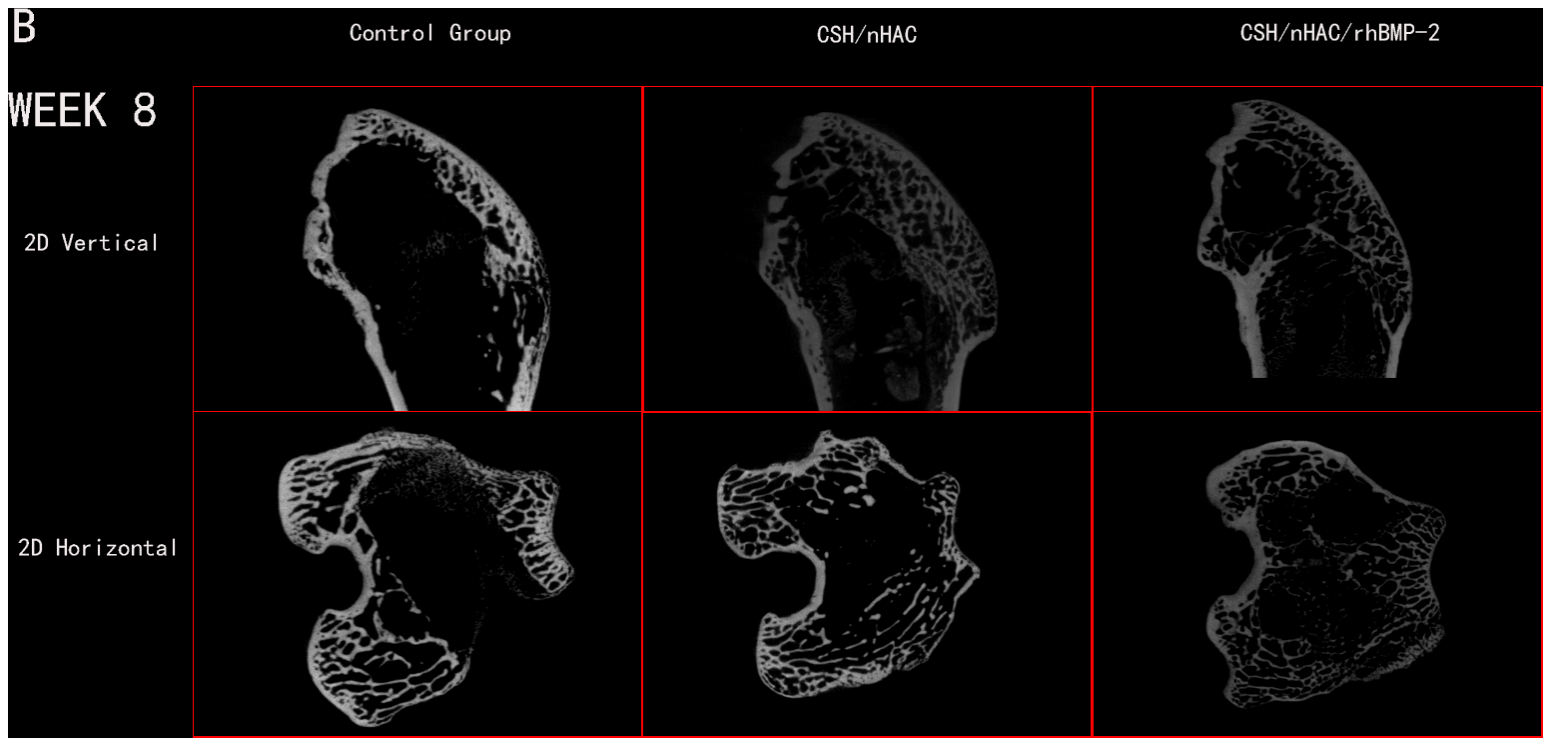
In vivo evaluation of the effects of CSH/nHAC scaffolds on new bone formation in a rabbit femoral condyle bone defect model. Treatment groups included: control group, CSH/nHAC group, CSH/nHAC/rhBMP-2 group. And the harvested Micro CT images for 8 weeks.

**Figure 8:**
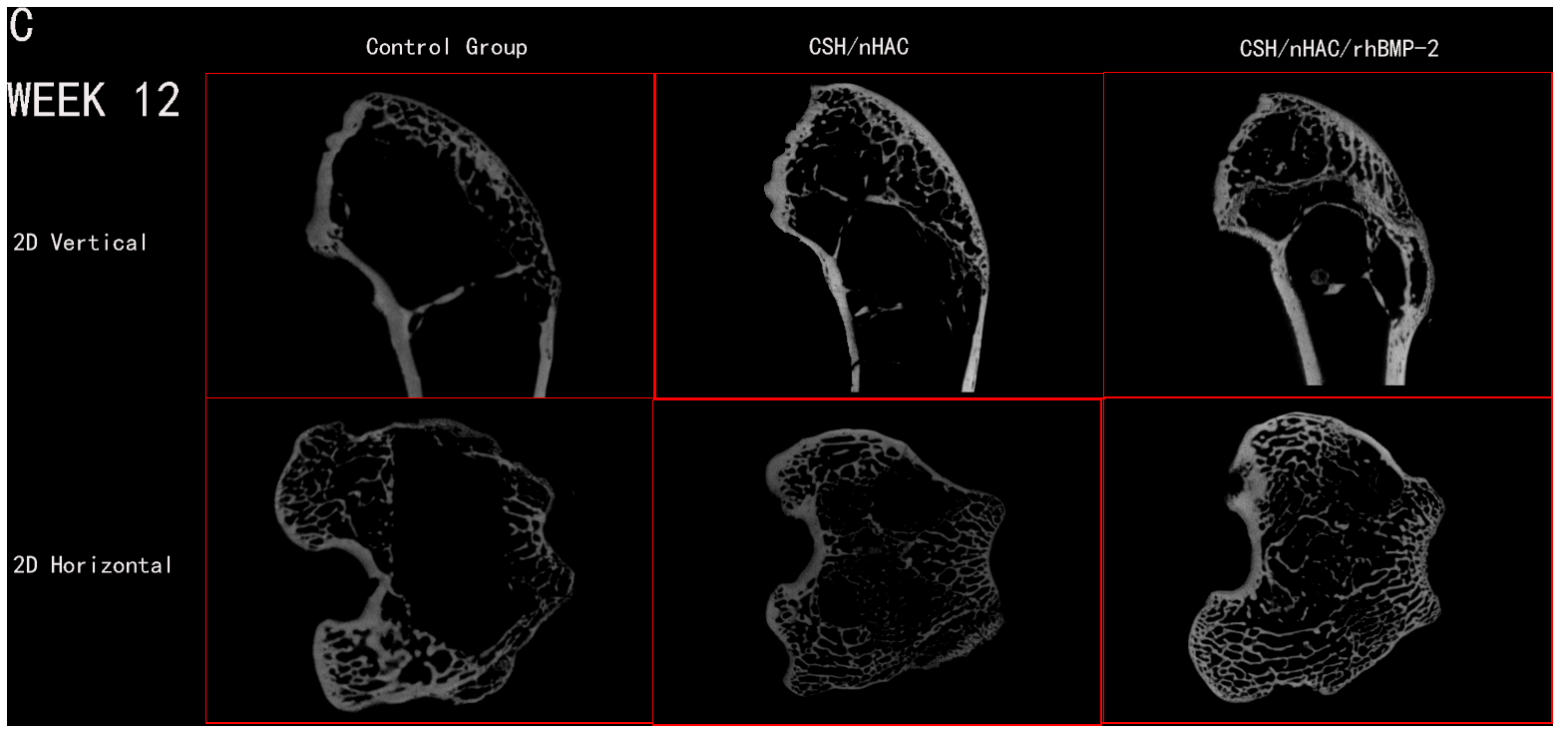
In vivo evaluation of the effects of CSH/nHAC scaffolds on new bone formation in a rabbit femoral condyle bone defect model. Treatment groups included: control group, CSH/nHAC group, CSH/nHAC/rhBMP-2 group. And the harvested Micro CT images for 12 weeks.

Micro CT reconstruction was performed for bone defect in the femoral condyle at predetermined time point as shown in the pictures below. In this study, the used defect model was a non-full-thickness distal femur cancellous bone defect. The newly bone formed mainly from the edge of defect and the surface of residual bone matrix. A layer of host bone cortical for better internal fixation was still reserved. Owing to the influence of host bone, use the reconstruction of Micro CT to distinguish the new bone formation tissue from host bone was almost impossible. The quantitative data about bone volume or bone parameter was also not accurate in the experiment. So the quantitative data about bone volume could not be obtained in the work. In order to observe the newly bone formation clearly, we use an auxiliary software of Micro CT to process the reconstruction image of femoral condyle. We got two kinds of images at last, the vertical image and the horizontal image. The defect treated with CSH/nHAC/rhBMP-2 had better bone formation tendency than the other two groups[Figure 6,7,8]. After surgery for 12 weeks, the bone defect was filled with newly bone tissue in group C, and less cancellous bone formed in group B. However, a clear bone defect can still be observed in group A [Figure 8].

### Histological Evaluation

**Figure 9:**
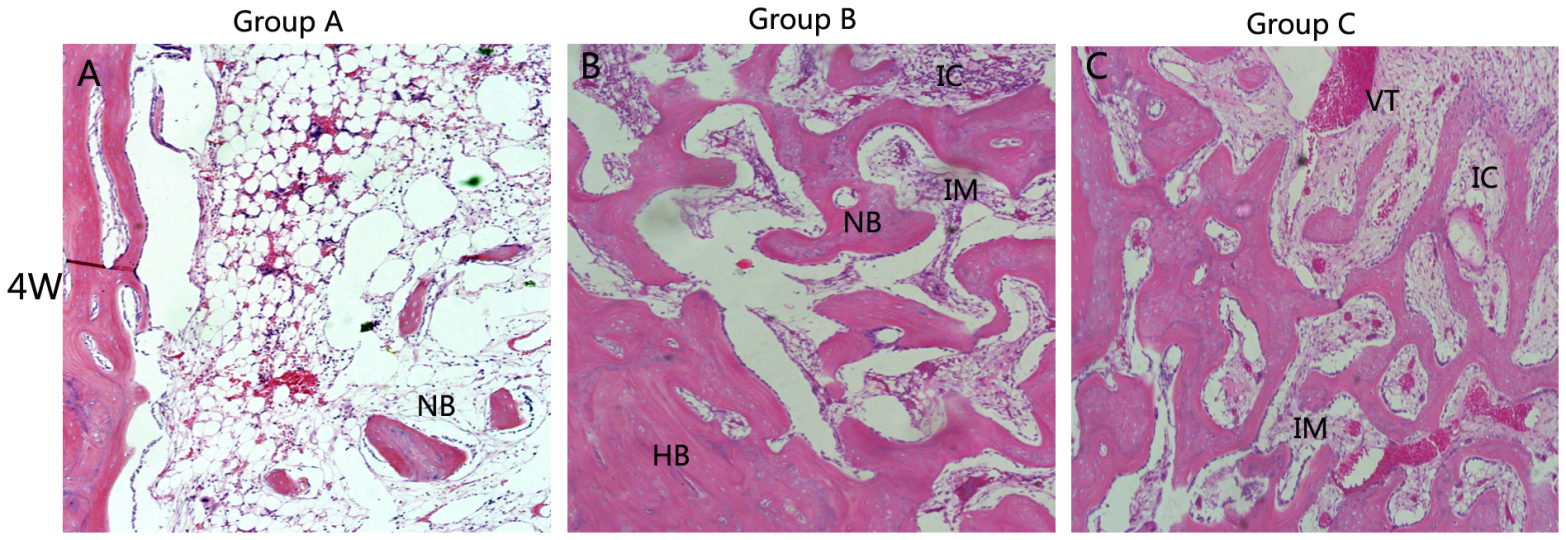
Histology photomicrographs of H&E staining of bone defects (10X) for three groups after 4 weeks. Abbreviations and signs used: newly bone (NB), host bone (HB), vascular tissue (VT), the implanted material (IM), bone marrow (BM), inflammatory cell(IC).

**Figure 10:**
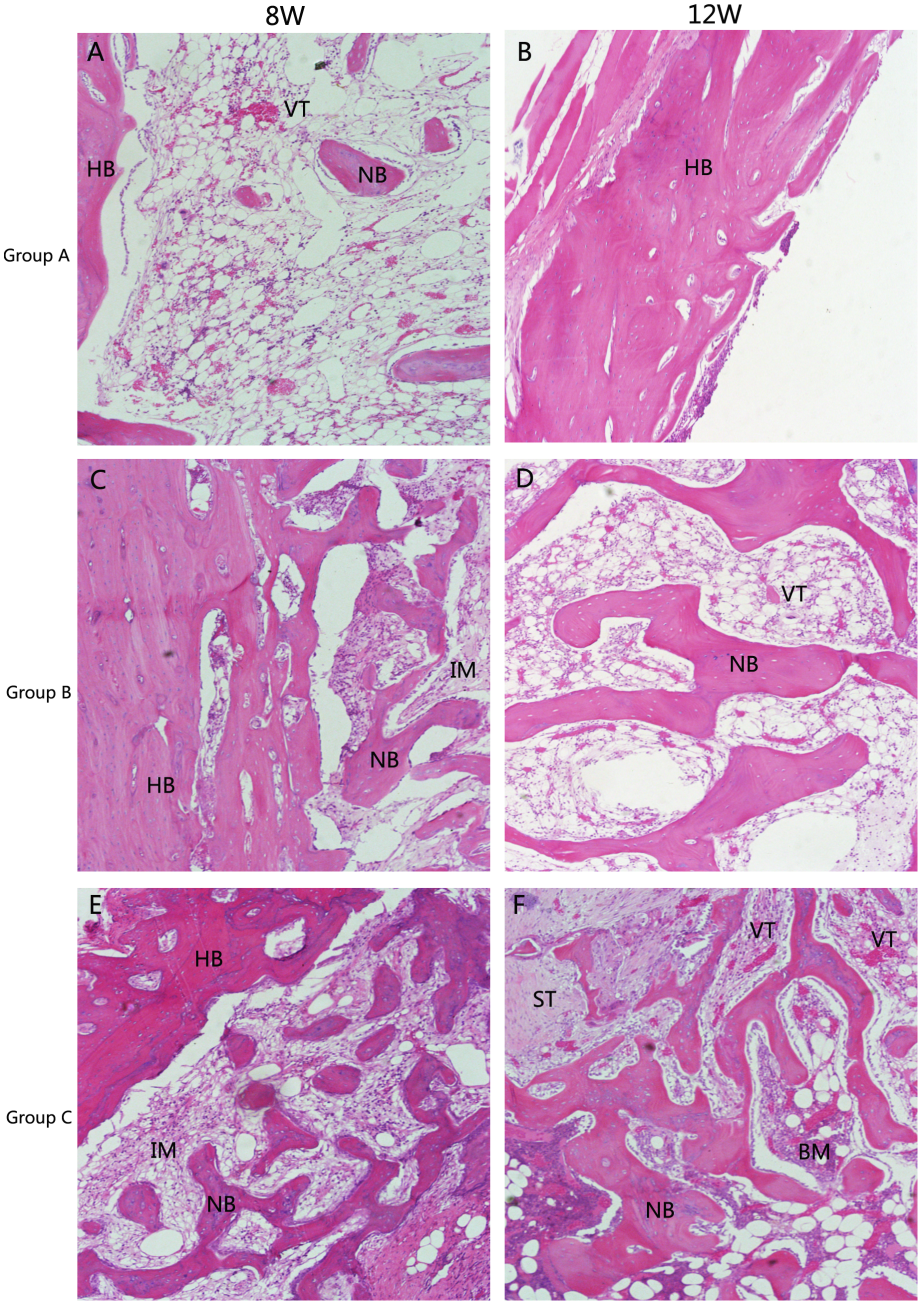
Histology photomicrographs of H&E staining of bone defects (10X) for three groups after 8 and 12 weeks. Abbreviations and signs used: newly bone (NB), host bone (HB), vascular tissue (VT), the implanted material (IM), bone marrow (BM), inflammatory cell(IC).

**Figure 11:**
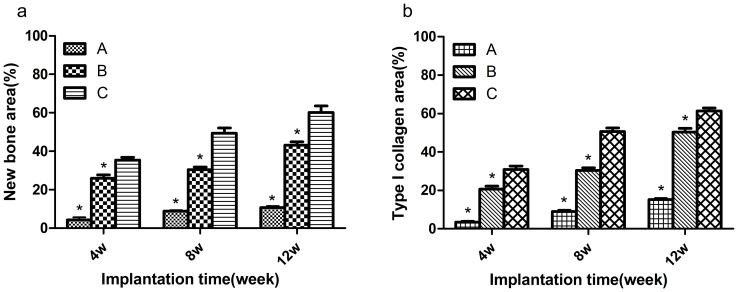
Histology photomicrographs of Sirius red staining of bone defects (10X) for three groups.

Micro CT images only disclose the newly formed mineralized bone [38]. Histological analysis was also performed to give a more detailed analysis on the new bone formation. Figure 9,10 showed the photographs of H$E staining sections after surgery. At 4 weeks, in group C, lots of osteocytes adhered to the implanted bone material and spread on it. Many osteoblasts and vessels assembled in the center area of bone defects. A thin layer of osteoid formed at the edge of the bone defect site. New trabeculae formed. In addition, the scaffolds partly degraded and divided into some small islands by the new formed bone tissue. The changes of group B were similar to group C, but there was less osteocyte, trabeculae and vessels compared with group C. However, few new bone tissues could be found in group A. On the other hand, some inflammatory cells can be seen in the center of bone defect area, indicating that a inflammatory response may be caused by the degradation of the implanted biocomposites[Figure 9]. At 8 weeks, more cells gathered at the interface between the implant materials and the new formed bone tissue. More trabeculae could be seen in the implanted section. At the same time, the scaffolds were further degraded. The residual materials were surrounded by areas of newly formed bone tissue. Compared with the samples at 4 and 8 weeks, the implant materials nearly degraded completely and were replaced by new trabeculae at 12 weeks. Most important of all, the quality of the new trabecular bone in group C was better than the other two groups. In group C, even some bone marrow presented around the newly formed bone and more blood vessel which stained red grew into the center area of bone defect, indicating that CSH/nHAC scaffold could be as a carrier of rhBMP-2 and it had a better capability of bone regeneration[Figure 10]. In the control group, the bone defect area was partly filled with new trabecular. Besides the qualitative results just as above described, the quantitative evaluation of bone regeneration capacity was also employed by calculating the percentage of new formed bone area on H&E staining sections at each time interval. From the results shown in Figure 11(a), in group C we concluded that the treated defect had the area of newly formed bone increased from 35.34±3.34% to 60.24±7.31% from 4 weeks to 12 weeks. In comparison, the Group B increased from 25.96±3.82% to 43.14±3.87%, and the control group increased from 4.32±2.98% to 10.70±1.72%. The results indicated that the injectable CSH/nHAC mixed with rhBMP-2 exhibited a better capability of osteogenesis compared with the other two groups(*p*<0.05).

**Figure 12:**
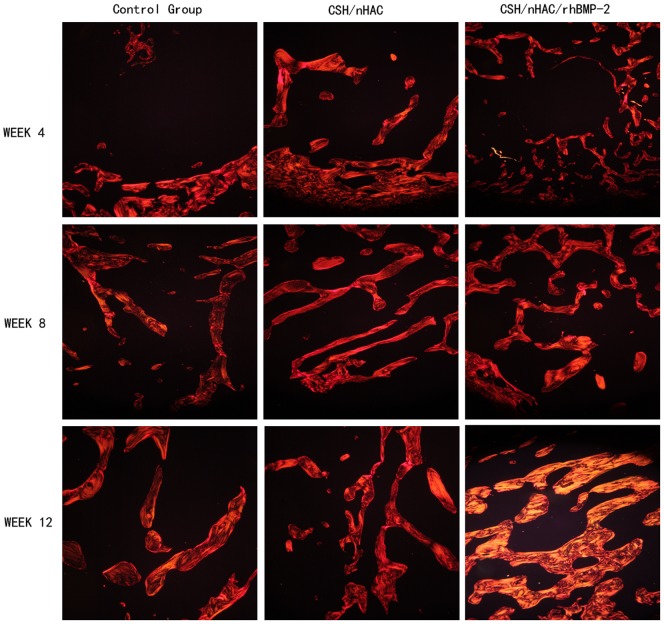
Quantitative analysis of H&E staining (a) and Sirius red staining (b) in these three groups (a). Error bars represent mean±SD for n = 3 (*p<0.05, compared with group C at the same time point).

The graphs of Sirius red staining displayed in Figure 12 also confirmed the obtained results from observation on the H&E staining sections. In Sirius red staining, bone trabecular was dyed in yellow or orange. At 4 weeks, in group A, only a little of type I collagen stained could be seen in the bone defect area. However, a large amount of type I collagen which was stained red or orange was clearly observed in the defect area at 4 weeks in the other two groups. Moreover, the qualitative results indicated that group C formed most type I collagen. After surgery for 8 and 12 weeks, we observed the same results[Figure 11(b)].

**Figure 13:**
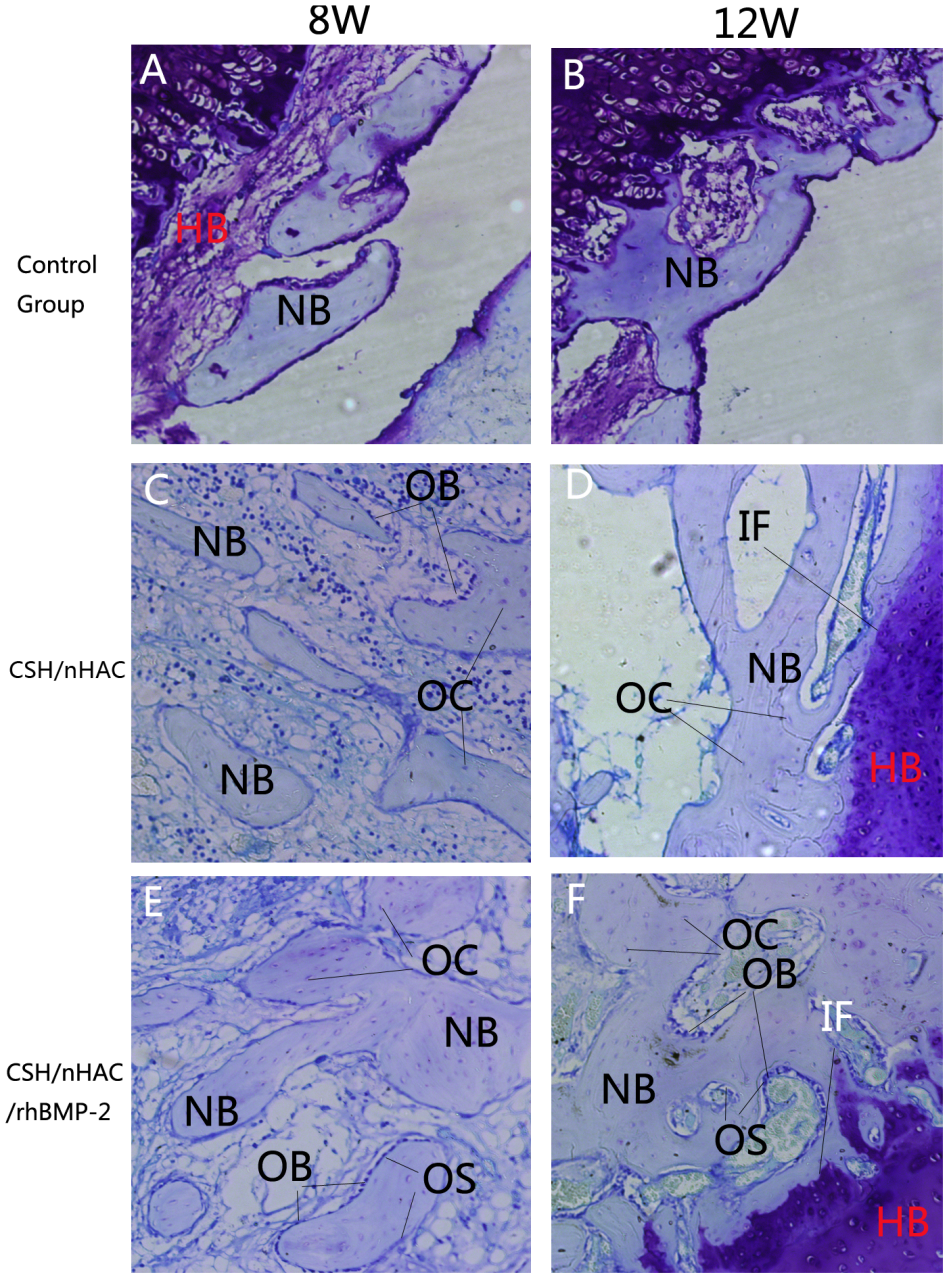
Histology photomicrographs of Toluidine blue staining of bone defects (10X). Abbreviations and signs used: newly bone (NB), host bone (HB), vascular tissue (VT), the implanted material (IM), newly osteoid (OS), bone marrow (BM), osteocyte(OC), osteoblast(OB), interface(IF).

The graphs of Toluidine blue staining displayed in Figure 13 also demonstrated the results observed above. In the Toluidine blue staining, we can observe the formation of osteiod which was not very clear in H&E staining. At 4 weeks, the newly formed mineralized bone of group B and C showed similar features. Very little of new formed bone could be seen in the bone defect area in the control group. After surgery for 8 weeks, a large proportion of new formed bone was dyed in light blue in group B and group C revealing that it was new formed bone tissue. In contrast, new bone trabecular was very loose in group A. At 12 weeks, the newly formed trabecular bone has gradually focused on most of the bone defect area in Group C, significantly better than the other two groups.

## Discussion

The results of this experiment indicated that the CSH/nHAC/rhBMP-2 were more bioactive than CHS/nHAC alone. The bioactivity of the composite was determined by the components of CSH and nHAC. During dissolution of CSH, calcium ions are released into the bone defect sites. Local increases in calcium ion concentration might affect the genesis and function of osteoblast, and they also may act a part in stimulating osteoblast differentiation [39]. Moreover, nHAC is mineralized fibrils assembled of type I collagen [40]. Both HA and collagen have been reported to enhance the process of osteoblast differentiation [41]–[43]. CSH and nHAC degraded at a different rate. During the degradation process, a bonelike apatite layer formed on the surface of the CSH/nHAC composite and it could improve bioactivity of the implanted materials [44]. Among all bone fiber collagen molecules, Type I collagen was considered as the most important kind of collagen fiber. It was considered to be a necessary gene of bone formation and remodeling, which can provide fiber reinforcement to the composite. So in this study, we choose Sirius red staining as an important pattern to observe the formation of type I collagen.

BMPs are members of the transforming growth factors β superfamily. The major biological effect of BMP lies in its ability to stimulate the aggregation, proliferation and differentiation of mesenchymal cells, which accelerates bone and cartilage formation. Among all the BMP family, BMP-2 has the strongest biological activity [15]. However, an important issue to consider in the use of BMPs is their side-effect [45]. BMPs are reported to be safe if they are used appropriately and the side effects include heterotopic ossification, local erythema and swelling, and immune response. Critical issues include the potential risk that BMPs will induce heterotopic bne formation, especially when implanted near to neural tissues. An unforseen issue is their role in osteoclast activation and formation. Osteoclasts are formed before osteoblasts which may lead to a weave of resorption that precedes the appearance and effect of osteoblasts [46]. And in this experiment, we choose rhBMP-2 as a growth factor. To achieve the optimum biological activity of rhBMP-2, the dose of rhBMP-2 was choosen as reported previously [47]. rhBMP-2 or other growth factors alone does not achieve the expected efficacy of bone or cartilage formation due to the short retention of protein in vivo, thus an ideal scaffold material as a delivery carrier is necessary. As a delivery carrier for growth factors, it should have two primary functions: first, it should maintain growth factors' bioactivity and optimal release amount at the implantation site to maximize the osteogenic effect of growth factor; second, it should be an osteoconducitve scaffold with suitable pore structure for vascularization and new bone formation [48]. Nowadays, a large number of natural and synthetic biomaterials have been fabricated for the economical delivery of rhBMP-2 [15], [49]. Inactive rat bone matrix loaded with rhBMP-2 has already been confirmed to heal a 0.5 cm segmental defect in rat femur [31]. rhBMP-2 has been shown to heal a 2.5 cm segmental defect in sheep femora, comparable with autograft in control animals [50]. Some studies revealed that CSH/nHAC was a promising scaffold for bone tissue engineering which exhibits better biocompatibility and bioactivity of bone regeneration [23], [44]. Moreover, there were very few reports about CSH/nHAC combined with rhBMP-2 in an animal bone defect model. In the in vitro study, an initial burst of BMP-2 from the CHS/nHAC scaffold was observed in the first day and approximately 37.5% of rhBMP-2 was released. After that, the rhBMP-2 was released in a sustained way. These results indicated that CSH/nHAC could be used as a delivery carrier for maintaining the release of rhBMP-2. Several possible reasons may account for the phenomena observed in the present study. Firstly, some relevant experiments indicate that rhBMP-2 exhibited a high level of affinity to HA. That is because rhBMP-2 has a highly repeating sequence of acidic amino acid Asp which has a chemical affinity to HA [51], [52]. CSH/nHAC materials fabricated in this experiment contained a quality of HA. The succeeding sustained release might have been caused by the degradation of the scaffold and the rhBMP-2 defused in to the SBF around with the implanted materials. Secondly, the initial burst release of rhBMP-2 in CSH/nHAC composite may be relevant with physical absorption [47]. Some relevant experiments reveal that the osteogenicity in the fast-release type was higher than in the slow-release type, indicating that the high initial release could induce more bone formation [53], [54]. The present experiment results also confirmed that the initial burst of rhBMP-2 led to more bone growth at the borders of the bone defect area, increasing the osteogenicity of scaffold surface through which the osteocyes could continue to spread on. The results of radiographic evaluation and histological examination confirm this result too. Critical bone defect was put forward firstly by Schmitz and it referred to the minimum bone defect which can not repair and heal spontaneous [55]. Some studies reported that in the rabbit femoral condyle a bone defect of 6 mm×8 mm can not heal itself, while other studies indicated that the critical bone defect in the rabbit femoral condyle was 5 mm×10 mm [56], [57]. In this study, a 7 mm×10 mmbone defect was created in the femoral condyle of rabbits. In the control group, after 12 weeks, no obvious bone formed in the defect, indicating that the bone defect area could not heal spontaneous. In the following work, further investigations will have to focus on large animal models or clinical applications and we will make a further study in comparison to commercial bone substitute and BMPs

## Conclusion

A new kind of injectable biocomposite was developed by introducing nHAC into CSH in this study. In vitro release kinetics and degradation characteristics of CSH/nHAC were studied. The bioactivity of CSH/nHAC with rhBMP-2 or without rhBMP-2 in vivo was examined. rhBMP-2 could improve the osteoinductivity of CSH/nHAC. The CSH/nHAC showed a gradual decrease in degradation with the content of nHAC increase. All these will enable the CSH/nHAC composite to develop a more promising hard tissue graft substitute for bone tissue engineering.
